# Managing ethical issues in community-based adolescent self-harm research. ethical practice in an adolescent self-harm preventative intervention research project with pupils in secondary schools in wales

**DOI:** 10.1192/j.eurpsy.2021.1547

**Published:** 2021-08-13

**Authors:** R. Parker

**Affiliations:** Decipher, Cardiff University, Cardiff, United Kingdom

**Keywords:** adolescent self-harm, preventative intervention research, secondary schools, ethics in research practice

## Abstract

**Introduction:**

Adolescent self-harm in Europe is a major public health challenge and shares a risk continuum with suicide. Recent research demonstrates a strong risk correlation with attempting suicide in the community-based adolescent self-harm population group, similar to research findings for populations in health setting-based research. In the UK increasing rates in hospital admissions represent the “tip of the iceberg” within the community, with potentially two thirds of the population group not accessing health services for support. This brings many health risks, including an increased suicide risk. Finding a solution to these issues requires a preventative intervention approach for young people, including community-based delivery to address service access barriers. Secondary schools are posited as key settings where this type of support could be delivered. But emerging UK research demonstrates challenges in completing adolescent self-harm research with young people in schools.

**Objectives:**

This paper centres on a current adolescent self-harm preventative intervention research project in Cardiff University. It focuses on ethical research practice in community-based adolescent self-harm research.

**Methods:**

This paper appraises some of the core ethical issues, challenges and their management in completing adolescent self-harm prevention intervention research in secondary school settings in Wales. It also provides an overview of the project’s innovative safety protocol design.

**Results:**

This project was successful in managing the potential risks to the school-based adolescent research participants.

**Conclusions:**

This work helps address some of the current research barriers to completing adolescent self-harm prevention intervention research in schools, to facilitate shared solutions to the urgent public health challenge of adolescent self-harm.

